# How Do Women Prepare for Pregnancy? Preconception Experiences of Women Attending Antenatal Services and Views of Health Professionals

**DOI:** 10.1371/journal.pone.0103085

**Published:** 2014-07-24

**Authors:** Judith Stephenson, Dilisha Patel, Geraldine Barrett, Beth Howden, Andrew Copas, Obiamaka Ojukwu, Pranav Pandya, Jill Shawe

**Affiliations:** 1 Reproductive Medicine, Institute of Women's Health, UCL, London, United Kingdom; 2 Health Sciences and Social Care, Brunel University, Uxbridge, United Kingdom; 3 Infection & Population Health, Institute of Epidemiology, UCL, London, United Kingdom; 4 University College London Hospitals, London, United Kingdom; University of Missouri, United States of America

## Abstract

**Main objective:**

To determine the extent to which women plan and prepare for pregnancy.

**Methods:**

Cross-sectional questionnaire survey of pregnant women attending three maternity services in London about knowledge and uptake of preconception care; including a robust measure of pregnancy planning, and phone interviews with a range of health care professionals.

**Main results:**

We recruited 1173/1288 (90%) women, median age of 32 years. 73% had clearly planned their pregnancy, 24% were ambivalent and only 3% of pregnancies were unplanned. 51% of all women and 63% of those with a planned pregnancy took folic acid before pregnancy. 21% of all women reported smoking and 61% reported drinking alcohol in the 3 months before pregnancy; 48% of smokers and 41% of drinkers reduced or stopped before pregnancy. The 51% of all women who reported advice from a health professional before becoming pregnant were more likely to adopt healthier behaviours before pregnancy [adjusted odds ratios for greatest health professional input compared with none were 2.34 (95% confidence interval 1.54–3.54) for taking folic acid and 2.18 (95% CI 1.42–3.36) for adopting a healthier diet before pregnancy]. Interviews with 20 health professionals indicated low awareness of preconception health issues, missed opportunities and confusion about responsibility for delivery of preconception care.

**Significance of the findings:**

Despite a high level of pregnancy planning, awareness of preconception health among women and health professionals is low, and responsibility for providing preconception care is unclear. However, many women are motivated to adopt healthier behaviours in the preconception period, as indicated by halving of reported smoking rates in this study. The link between health professional input and healthy behaviour change before pregnancy is a new finding that should invigorate strategies to improve awareness and uptake of pre-pregnancy health care, and bring wider benefits for public health.

## Introduction

The period before conception is increasingly regarded as important for the health of pregnant women and future generations. Successive reports from the Centre for Maternal and Child Enquiries [6], [5] conclude that lack of preconception care is a contributory factor in maternal deaths, while evidence from life course epidemiology [18], [2] and epigenetics [15] highlights the importance of the intrauterine environment in determining chronic disease risk in childhood and adulthood. Factors such as maternal diet and nutritional status, which can be modified before conception, have an important influence on the intrauterine environment and fetal development. Consequently, the preconception period is seen as a critical period where intervention can lead to both short term benefit, by reducing pregnancy complications and adverse birth outcomes, and long term health gain, as emphasised in the WHO Global Action Plan for the Prevention and Control of Non-communicable Diseases 2013–2020 [34].

There is a fair degree of consensus among expert bodies and in professional guidelines about what preconception care should entail, particularly in the USA. It includes folic acid supplementation for all women to prevent neural tube defects [12], reduce preterm birth [4] and congenital heart defects [14]; stopping smoking, reducing alcohol consumption, achieving or maintaining a healthy weight and screening for infection [13]. Environmental and occupational hazards have also been identified [13]. For women with chronic conditions, such as diabetes, and particularly for women taking potentially teratogenic medication (e.g. angiotensin-converting enzyme inhibitors, which are increasingly used to control blood pressure in younger women, anticonvulsants, isotretinoin for acne and statins) additional intervention and review of medication is required [9].

However, despite the importance attributed to good pre-pregnancy care, there is little understanding of women's behaviour or the information they acquire in preparation for pregnancy, or how this relates to uptake of care or interaction with health care professionals. Government policy in UK and USA aims to reduce perinatal morbidity and mortality by promotion of preconception care [10], but this requires an awareness of preconception health and care among both the public and health professionals and involves pregnancy planning on the part of the woman and health services before conception. These areas have not been well researched. We, therefore, assessed how women prepare for pregnancy through a survey of nearly twelve hundred women attending maternity services in North London. We assessed the level of information acquired about preconception health and care, the nature and extent of their preparation for pregnancy, and the likely impact of health professional input on positive behaviour change before conception. We also assessed the views and engagement of health professionals with preconception care through qualitative interviews with doctors and nurses from a range of health care professions.

## Materials and Methods

### Ethics statement

The survey was approved by the National Research Ethics Service, NRES Committee London- Bromley (REC reference 11/LO/0881). The approval was given as part of a larger study of preconception health and care in England. In accordance with standard practice approved by the research ethics committee, women gave consent by opting to complete the questionnaire. Women who agreed to follow up gave explicit signed consent.

### Conduct of the survey

The antenatal survey was conducted between November 2011 and May 2012 in the maternity services of three North London Hospitals, which were selected to enable women from diverse ethnic and socioeconomic backgrounds to participate. Women attending these maternity services represent a mix of both low and high risk pregnancies.

Women were approached by trained researchers. They were given an information leaflet about the project and the consent process, and invited to consent to completing the antenatal questionnaire, and being contacted for follow-up questionnaire or interview. Women who did not wish to be followed-up were invited to complete the baseline questionnaire. For practical reasons, the recruitment process varied by site, but the aim in all three hospitals was to recruit women early in pregnancy to reduce recall bias and to recruit from both low and high risk clinics. Women filled in a self-completion pen-and-paper questionnaire while they were waiting for their appointment. The data was entered onto computer by a commercial data entry company (Abacus).

### Design of the questionnaire

We carried out a literature review to explore themes and topics that should be covered in the questionnaire and examined preconception care questionnaires used in the Southampton Women's Survey [20] and in Sweden [32], to design an appropriate study questionnaire about health and health-related behaviour before and during pregnancy. The questionnaire was co-developed with the Margaret Pyke Forum, a user group that includes members of the public and patients of various ages and backgrounds, and then piloted with five pregnant women attending a maternity service in London.

The questionnaire asked whether respondents had visited a health professional (GP, nurse, midwife, consultant, pharmacist, family planning or antenatal professional) to obtain advice on getting pregnant; whether they had accessed information by any means about folic acid and vitamin supplements and a list of eleven other preconception health behaviours (healthy diet, healthy weight, alcohol, smoking, immunisations, recreational drugs, STIs, dental checks, caffeine, stopping contraception/fertility advice) from a health professional, from family or friends, or other sources including finding out for themselves. Health professional input was categorised on three levels: none; visiting a health professional and receiving advice on any of the eleven preconception health behaviours listed above *or* on folic acid supplementation (‘partial’ advice); and receiving advice from a health professional on any of the eleven listed preconception health behaviours *and* on folic acid supplementation (‘full’ advice). To assess the extent of pregnancy planning, we used the London Measure of Unplanned Pregnancy, (LMUP), a six-item self-completion questionnaire with established psychometric properties that scores pregnancy planning or intention from 0–12 [1]. Scores of 0 to 3 were categorised as ‘unplanned,’ 4 to 9 as ‘ambivalent’ and 10 to 12 as ‘planned.’ Women were categorised as having a ‘relevant medical condition’ if they reported, in the three months before pregnancy any condition or treatment that we identified from review of the literature with potential for adverse interaction with pregnancy and where medical review of the condition and/or treatment before pregnancy would be advised. Our literature review produced a long list of such conditions, including common disorders like diabetes and obesity as well as rare conditions requiring specialist review and management.

### Sample size calculation

We determined the sample size by selecting at least 80% power to detect differences that we consider of practical importance at the 5% significance level in the prevalence of a range of key outcomes (e.g. folic acid consumption) where the prevalence may vary from low (e.g. 5–10%) to more common (e.g. 50%). Specifically we noted that a sample size of 948 participants provides 80% power to detect as significant a difference in the prevalence of an outcome between two groups of equal size (e.g. grouped by ethnicity or other factors) of 5% versus 10%, and 824 participants provides 80% power for a difference of 45% versus 55%. We therefore set a minimum sample size of 1000 participants so as to have more than 80% power for important differences across a range of outcomes of interest.

### Statistical approach to analysis of survey data

The chi-squared test was used to test associations between participant characteristics and measures of information acquired, professional input, behaviour change and folic acid consumption. All factors are treated as categorical and the categorisations used for the tests are those presented in the tables. Logistic regression was used to calculate unadjusted and adjusted odds ratios for a range of behaviour change outcomes and folic acid consumption, which are presented with 95% confidence intervals. The key explanatory factor investigated in the regressions is level of health professional input considered in 3 categories, whilst other participant characteristics are viewed as potential confounders. The set of potential confounders adjusted for to generate adjusted odds ratios is common to all outcomes and is all those factors found significantly associated with any of the behaviour change outcomes or with folic acid consumption in univariate analysis. Adjustment for age and LMUP score is made treating these as continuous factors, whilst other factors are treated as categorical with categories as presented in the tables.

### Interviews

We aimed to recruit a purposive sample of 20 health professionals from all nine governmental regions of England working in general practice, obstetrics & gynaecology, midwifery and sexual & reproductive health. All participants were contacted by email and invited to either face-to-face or telephone interview. The interviews explored how policy, guidelines and recommendations are implemented in day-to-day practice and identified perceived barriers. We used a topic guide consisting of a series of questions about what constitutes preconception care, the nature of current provision in their services, who should have responsibility for providing preconception care, to whom, the perceived barriers to service delivery and how the current situation could be improved. The interviews were carried out by four researchers between August 2011 and July 2012. They were tape-recorded and transcribed *verbatim*. There were no explicit time constraints applied, but the interviews lasted between 15 and 40 minutes. Data were analysed thematically, according to the principles of qualitative description [31], [26]. Further information about how the conduct of the interviews met the consolidated criteria for reporting of qualitative research (COREQ) is shown in appendix S1.

## Results

### Antenatal survey

We recruited 1173 of 1288 women invited to take part at the three study sites, giving an overall response rate of 90% (86%, 91% and 94% at each of the three hospitals).

Table 1 shows the level of information about preconception health and extent of health professional input by demographic characteristics, pregnancy history and current pregnancy planning (using the London Measure of Unplanned Pregnancy). One quarter (25%) of women reported a medical condition in the three months before becoming pregnant that might affect the pregnancy and 30% of all women had had a previous miscarriage, stillbirth or termination due to fetal abnormalities. 72% of women were aged 30 or above and nearly three-quarters (73%) of women reported a planned pregnancy. Despite the high level of pregnancy planning in this survey, and previous miscarriage, stillbirth or termination for fetal abnormalities, 34% of all women reported acquiring no information about eleven preconception health behaviours and 49% reported no input from a health professional about preconception care (Table 1). Just over half (51%) of all women, and fewer than two thirds (63%) of women with planned pregnancies, took folic acid before pregnancy (Table 2).

**Table 1:** Information acquired about preconception health and level of health professional input before pregnancy.

Characteristic	% (n)	Extent of information acquired (any source) about 11 topics on preconception health			Level of health professional input before pregnancy		
		***None***	***1–6 topics***	***7–11 topics***	***None***	***Partial Advice*** *	***Full Advice*** **
	All	34 (404)	42 (487)	24 (282)	49(573)	29(344)	21 (250)
Age		*P = 0.04*			*P = 0.22*		
** <30**	28 (288)	41 (118)	40 (116)	19 (54)	56(161)	26 (74)	18 (52)
** 30–34**	41 (419)	32 (132)	42 (178)	26 (109)	48(202)	29(122)	22 (94)
** 35+**	31 (324)	31 (102)	44 (142)	25 (80)	48(153)	30 (98)	22 (71)
Ethnic group		*P = 0.02*			*P = 0.89*		
** White**	68 (705)	32 (223)	44 (307)	25 (175)	49 (346)	29(203)	22 (154)
** Mixed**	6 (58)	40 (23)	40 (23)	21 (12)	53 (31)	29 (17)	17 (10)
** South Asian**	12 (120)	33 (39)	43 (51)	25 (30)	45 (54)	32 (38)	23 (28)
** Black**	10 (101)	51 (52)	31 (31)	18 (18)	55 (54)	24 (24)	20 (20)
** Other**	6 (59)	25 (15)	51 (30)	24 (14)	54 (32)	25 (15)	20 (12)
Place of birth		*P = 0.55*			*P = 0.02*		
** Outside UK**	52 (539)	33 (177)	44 (238)	23 (124)	46 (245)	32(171)	22 (120)
** UK**	48 (493)	34 (167)	41 (202)	25 (124)	54 (266)	26(127)	20 (99)
Employment status		*P = 0.01*			*P = 0.28*		
** Employed or full-time education**	68 (714)	30 (214)	44 (313)	26 (187)	51 (365)	28(196)	21 (151)
** Unemployed**	10 (101)	42 (42)	36 (36)	23 (23)	40 (40)	33 (33)	27 (27)
** At home or on maternity leave**	19 (194)	40 (77)	41 (80)	19 (37)	46 (88)	31 (60)	23 (44)
** Other**	4 (39)	41 (16)	49 (19)	10 (4)	54 (21)	33 (13)	13 (5)
Highest educational achievement		*P = 0.008*			*P = 0.36*		
** Degree**	66 (682)	30 (205)	45 (306)	25 (171)	49 (336)	29(199)	21 (144)
** Dip/NVQ3/A-level**	18 (183)	37 (68)	40 (73)	23 (42)	49 (89)	26 (48)	25 (46)
** Other qualifications**	13 (133)	44 (58)	32 (43)	24 (32)	54 (71)	27 (35)	19 (25)
** No qualifications**	3 (30)	37 (11)	57 (17)	7 (2)	33 (10)	43 (13)	23 (7)
Relevant medical condition		*P = 0.47*			*P = <0.001*		
** No**	75(883)	35(310)	42(243)	19(205)	53(466)	28(243)	19(170)
** Yes**	25(290)	32(94)	41(119)	27(77)	37(107)	35(101)	28(80)
Taking medication		*P<0.001*			*P = <0.001*		
** No**	74 (863)	38 (324)	40 (349)	22 (190)	53 (454)	29(249)	18 (156)
** Yes**	26 (310)	26 (80)	45 (138)	30 (92)	39 (119)	31 (95)	31 (94)
Previous live birth		*P = 0.004*			*P = 0.002*		
** No**	59 (607)	30 (184)	45 (273)	25 (150)	50 (300)	25(154)	25 (151)
** Yes**	41 (428)	40 (172)	39 (166)	21 (90)	51 (219)	32(137)	16 (70)
If previous live birth, any conditions/abnormalities		*P = 0.91*			*P = 0.18*		
** No**	64 (227)	40 (90)	40 (91)	20 (46)	52 (117)	34 (76)	14 (31)
** Yes**	36 (126)	39 (49)	39 (49)	22 (28)	46 (58)	33 (41)	21 (27)
Previous miscarriage, stillbirth or termination		*P = 0.09*			*P = 0.02*		
** No**	70 (654)	36 (236)	41 (267)	23 (151)	53(345)	27(175)	20(131)
** Yes**	30 (279)	29 (81)	47 (132)	24 (66)	43(119)	32(88)	25(70)
Pregnancy Intention		*P<0.001*			*p>0.001*		
** Unplanned**	3 (36)	61 (22)	11 (4)	28 (10)	66(23)	23(8)	11(4)
** Ambivalent**	24 (272)	53 (144)	33 (90)	14 (38)	62(166)	28(76)	10(27)
** Planned**	73 (840)	27 (230)	46 (385)	27 (225)	45(375)	30(252)	25(211)

*Includes advice on folic acid or other preconception health behaviours

**Includes advice on folic acid and other preconception health behaviours.

**Table 2:** Health behaviour change and supplement consumption before pregnancy.

	Behaviour change			Supplement consumption		
Characteristic %(n)	*Reduced smoking*	*Reduced Alcohol*	*Changed diet*	*No Folic Acid*	*Folic Acid only*	*Folic Acid and Multi Vitamins*
**Total**	48 (115)	41 (262)	31 (346)	49(572)	26(301)	25(285)
Age	*P = 0.003*	*P = 0.21*	*P = 0.007*	*P<0.001*		
** <30**	31 (21)	33 (36)	24 (67)	66(187)	24(68)	10(29)
** 30**–**34**	56 (50)	41 (113)	32 (131)	43(178)	27(110)	30(125)
** 35+**	57 (37)	43 (91)	36 (114)	45(205)	27(123)	28(131)
Ethnic group	*P = 0.89*	*P = 0.96*	*P = 0.27*	*P<0.001*		
** White**	51 (85)	41 (198)	32 (219)	44(306)	28(196)	28(194)
** Mixed**	45 (5)	36 (10)	29 (17)	57(32)	23(13)	20(11)
** South Asian**	44 (7)	37 (14)	35 (42)	55(66)	20(24)	25(30)
** Black**	47 (7)	36 (8)	23 (23)	70(69)	17(17)	12(12)
** Other**	36 (4)	40 (12)	24 (14)	46(27)	24(14)	30(18)
Place of birth	*P = 0.23*	*P = 0.76*	*P = 0.83*	*P = 0.43*		
** Outside UK**	43 (43)	40 (106)	31 (165)	49(264)	27(141)	24(130
** UK**	52 (63)	41 (137)	31 (152)	47(230)	25(120)	28(135)
Employment status	*P = 0.002*	*P = 0.69*	*P = 0.07*	*P = 0.001*		
** Employed or full-time education**	56 (80)	41 (192)	33 (236)	45(315)	27(189)	28(202)
** Unemployed**	15 (4)	31 (9)	22 (22)	63(63)	23(23)	14(14)
** At home or on maternity leave**	45 (18)	39 (35)	27 (51)	55(110)	25(50)	20(39)
** Other**	50 (5)	45 (10)	28 (11)	37(11)	26(8)	37(11)
Highest educational achievement	*P = 0.02*	*P = 0.03*	*P = 0.03P = 0.03*	*P<0.001*		
** Degree**	58 (69)	43 (201)	33 (226)	39(266)	29(295)	32(214)
** Dip/NVQ3/A-level**	44 (22)	32 (24)	29 (53)	62(111)	18(33)	20(37)
** Other qualifications**	35 (14)	30 (17)	28 (35)	70(90)	20(26)	10(12)
** No qualifications**	17 (1)	0 (0)	10 (3)	63(19)	30(9)	7(2)
Relevant medical condition	*P = 0.99*	*P = 0.63*	*P = 0.06*	*P = 0.07*		
** No**	48(81)	40(193)	29(246)	49(500)	26(267)	25(264)
** Yes**	48(34)	42(69)	35(100)	57(72)	27(34)	16(21)
Taking medication	*P = 0.82*	*P = 0.53*	*P = 0.51*	*P<0.001*		
** No**	48 (87)	40 (175)	30 (247)	53(450)	26(217)	21(183)
** Yes**	47 (28)	43 (87)	32 (99)	40(122)	27(84)	33(102)
Previous live birth	*P = 0.01*	*P<0.001*	*P = 0.09*	*P = 0.46*		
** No**	55 (76)	45 (176)	33 (196)	48(281)	26(159)	26(159)
** Yes**	37 (29)	29 (61)	28 (116)	51(216)	25(106)	24(103)
If previous live birth, any conditions/abnormalities	*P = 0.87*	*P = 0.24*	*P = 0.82*	*P = 0.03*		
** No**	40 (17)	32 (39)	29 (64)	45(99)	27(60)	28(63)
** Yes**	38 (10)	23 (12)	28 (35)	58(73)	24(30)	18(22)
Previous miscarriage, stillbirth or termination due to abnormalities	*P = 0.06*	*P = 0.01*	*P = 0.02*	*P = 0.02*		
** No**	54 (67)	38 (154)	29 (187)	50(322)	26(166)	24(158)
** Yes**	41 (32)	49 (73)	37 (101)	41(112)	27(76)	32(88)
Pregnancy Intention	*P<0.001*	*P<0.001*	*P<0.001*	*P<0.001*		
** Unplanned**	10 (1)	13 (1)	0 (0)	94(33)	3(1)	3(1)
** Ambivalent**	17 (14)	12 (15)	12 (31)	81(212)	12(32)	7(19)
** Planned**	68 (99)	49 (245)	38 (314)	37(312)	31(259)	32(263)

Smoking before pregnancy was reported by 21% of women (5% passive only, 10% smoking 0–10 cigarettes per day and 7% smoking more than 10 per day) but nearly half of them (48%, Table 2) reported reducing or stopping smoking before pregnancy. 61% of women reported drinking 1 or more units of alcohol per week (33% reporting 0–4 units and 28% more than 4 units per week) and about two fifths of them (41%, Table 2) reduced or stopped drinking alcohol before pregnancy. Reductions in both smoking and alcohol were associated with pregnancy planning, educational achievement and having a previous live birth (Table 2). Reducing or stopping smoking before pregnancy was also significantly associated with age and employment status, while reducing or stopping alcohol was significantly associated with having a relevant medical condition or a previous miscarriage, stillbirth or termination due to abnormalities (Table 2). Findings were similar for the women (31%) who reported changing to a healthier diet before they became pregnant. Taking folic acid or vitamin supplementation before pregnancy was significantly associated with age, ethnicity, employment status and educational achievement, taking (other) medication, having a previous miscarriage, stillbirth or termination due to fetal abnormality and pregnancy planning (Table 2).

Overall, 27% of women reported visiting a health professional for advice about getting pregnant although their source of information on a range of preconception health behaviours was more commonly outside the health profession (Table 3). In contrast to information acquired about pre-pregnancy health behaviours, getting advice from a health professional before pregnancy was not significantly associated with employment status or educational attainment (Table 1). While both information acquired and health professional input were associated most strongly with a previous live birth, taking medication and planning a pregnancy, extent of information acquired was also significantly associated with age, educational achievement, ethnic group, and employment status, while health professional input was significantly associated with being born in the UK or having a relevant medical condition.

**Table 3:** Source of advice on supplements and health behaviours received before pregnancy.

Supplements	GP	Health Professional	Family/Friends	Other*
**Folic acid**	28.6(336)	6.1(72)	24.4(286)	17.2(202)
**Multivitamins for women trying to get pregnant e.g. Pregnacare**	6.4(75)	3.2(37)	10.7(125)	11.8(138)
**Ordinary multivitamins**	1.1(13)	0.3(3)	2.0(24)	2.8(33)
**Vitamin D**	3.8(45)	1.8(21)	2.0(23)	3.1(36)
**Iron**	3.4(40)	0.9(11)	2.4(28)	3.3(39)
**Omega 3**	1.4(17)	0.9(11)	4.1(48)	4.4(52)
**Vitamin C**	2.0(23)	0.7(8)	2.4(28)	3.8(44)
**Zinc**	0.5(6)	0.7(8)	0.9(11)	2(23)
Other preconception health behaviours				
**Eating a healthy diet**	16.1(189)	5.2(61)	22.5(264)	24.8(291)
**Healthy weight**	10.0(117)	3.9(46)	5.1(60)	15.0(176)
**Caffeine**	8.7(102)	4.4(52)	13(152)	22.2(260)
**Alcohol**	13.3(156)	5.0(59)	15.3(180)	26.3(309)
**Smoking**	13.1(154)	4.5(53)	13.6(160)	23.5(272)
**Street drugs**	6.9(81)	3(35)	7.6(89)	16.4(192)
**Immunisations**	6.9(81)	2.7(32)	3.6(42)	9.0(106)
**Stopping contraception**	9.4(110)	2.9(34)	8.2(96)	13.7(161)
**Conception/fertility advice**	10.0(117)	5.0(59)	6.7(79)	12.4(146)
**Dental Care**	5.8(68)	2.0(23)	5.5(64)	9.5(111)
**STI Check**	7.6(89)	3.2(38)	3.6(42)	10.4(122)

*Includes finding out themselves, internet and books etc.

One quarter of the sample reported a relevant medical condition: diabetes, epilepsy, hypertension, HIV, kidney disease, lupus, thyroid disease, sickle cell, obesity, acne, asthma, depression, rheumatoid arthritis or lung disease and/or a treatment with potential teratogenic effects (Table 1). The most common conditions were obesity (5%), asthma (5%), polycystic ovary syndrome (5%) and depression/anxiety (4%). The level of pregnancy planning (66% planned, 30% ambivalent and 4% unplanned) among women with a relevant medical condition was no greater than that of women with no relevant medical condition (72% planned, 22% ambivalent and 3% unplanned, p = 0.14). Women with a relevant medical condition were more likely than other women to report input from a health professional before pregnancy, but not more information about pre-pregnancy health (Table 1) or greater change in health behaviours before pregnancy (Table 2).

Table 4 shows associations between health professional input and pre-pregnancy behaviour change: cutting smoking, alcohol, changing to a healthier diet and taking folic acid supplements. All four positive health behaviour changes before pregnancy were associated in a dose-dependent fashion with level of input from health professionals; the association with changing to a healthier diet and taking folic acid remained statistically significant after adjusting for other factors associated with pre-pregnancy behaviour change including age, ethnicity, education, previous live birth, medications, previous miscarriage/stillbirth, and LMUP score. Table 1 shows that, in contrast to information acquired, health professional input was not significantly associated with employment or educational attainment.

**Table 4:** Associations between health professional input and change in health behaviours before pregnancy and folic acid consumption.

	Level of health professional input			
Outcome	None	Includes advice on folic acid or other preconception health behaviours	Includes advice on folic acid and other preconception health behaviours	**p-value**
**Reduced alcohol intake**				
** % (n)**	30 (95)	44 (79)	62 (88)	
** OR (95% CI)**	1 -	1.86 (1.27–2.72)	3.77 (2.50–5.71)	**<0.001**
** Adjusted** 1 **OR (95% CI)**	1 -	1.32 (0.77–2.24)	1.45 (0.84–2.52)	**0.36**
**Reduced smoking**				
** % (n)**	37 (43)	55 (37)	63 (35)	
** OR (95% CI)**	1 -	2.09 (1.14–3.86)	2.83 (1.46–5.47)	**0.003**
** Adjusted** 1 **OR (95% CI)**	1 -	1.83 (0.67–5.04)	0.89 (0.31–2.51)	**0.42**
**Healthier diet**				
** % (n)**	20 (112)	37 (123)	46 (110)	
** OR (95% CI)**	1 -	2.33 (1.72–3.16)	3.41 (2.45–4.73)	**<0.001**
** Adjusted** 1 **OR (95% CI)**	1 -	2.42 (1.62–3.61)	2.18 (1.42–3.36)	**<0.001**
**Folic acid consumed**				
** % (n)**	37 (209)	55 (189)	76 (188)	
** OR (95% CI)**	1 -	2.09 (1.59–2.75)	5.31 (3.79–7.44)	**<0.001**
** Adjusted** 1 **OR (95% CI)**	1 -	1.65 (1.12–2.44)	3.86 (2.35–6.35)	**<0.001**

1 Adjusted for age, ethnicity, education, previous live birth, medications, previous miscarriage/stillbirth, and LMUP score.

### Interviews

We interviewed 21 health professionals by telephone: four consultants in obstetrics and gynaecology, eight community based consultants (or clinical leads) in sexual and reproductive health, seven general practitioners, one sexual health specialist nurse and one midwife. The key findings related to awareness of guidelines about preconception health and care, responsibility for providing pre-pregnancy care, and existing barriers to provision.

Most of the health professionals we interviewed were able, when asked, to define pre-pregnancy care as counseling women on factors such as exercise, smoking cessation, and reducing alcohol to encourage a healthy pregnancy. One interviewee responded with an interesting, but significant, misinterpretation:

*“I don't do pre-pregnancy care – I tend to leave that for the antenatal appointments” (Interview 15, GP).*

Most interviewees expressed only a vague awareness of guidelines on pre-pregnancy health and care, although a number were able to identify potential sources of information, such as the Department of Health, RCOG, and NICE websites. Some interviewees expressed the wish for guidelines specific to preconception care that would lead to more uniformity in their practice.

While most respondents felt that preconception care was important for optimising pregnancy and birth outcomes, there was a general sense that it was someone else's responsibility (see Interviews 1). Only two interviewees, both clinical leads from sexual and reproductive health, thought that preconception care was definitely within their role and should be part of routine contraceptive care, e.g.:

*“… I think somebody like myself, is in quite a good position to keep doing general education every time you see somebody for condoms or for the pill, to keep saying, look if you're ever to think about having a baby, you should be thinking about this. […] I would try and funnel in some advice and drop in a few thoughts about folic acid, thought about getting your diabetes under control” (Interview 4, Clinical lead).*

#### 

Interviews 1: Professional responsibility for pre-pregnancy care

*“I feel this is not something that resources should be going into. I think it's not a professional area, I think this is about people being educated and informed through the media of how to keep healthy and that they. that is the message that they need to have” (Interview 11, Clinical lead)*

*“And it wouldn't necessarily be with a GP, would it, it could be with the practice nurse or it could even be with a midwife, I mean that might be a way forward, if there was any money, that you could have midwives involved both in pre-pregnancy and postnatal care” (Interview 9, Clinical lead).*

*“. but I don't think it's all down to midwives, I think it's got to be the whole health service in general” (Interview 21, midwife).*

*“…it depends on where… is this a job of the medical profession? Or is it a job of the education services? They overlap don't they?” (Interview 13, GP)*

*“Well, it's not specifically part of our service specification so it's not something that we're required to do under the service spec as it were …” (Interview 5, Clinical lead).*

Most interviewees felt there should be a targeted public health campaign to promote pre-pregnancy health, and there was a view that women should be encouraged to take the initiative themselves to obtain information about preconception health (see Interviews 2). Specialist preconception services were seldom regarded as the solution.

#### 

Interviews 2: Information and Education

*“Publicity campaigns about ways that your health behaviours could impact on your pregnancy would be very useful so that people get their information through their media that they would use anyway rather than through a … what is perceived as a health service which tends to be used as an illness service in this country” (Interview 11, Clinical lead).*

*“You might get more engagement through the media than you would through health professionals. If you ran a story on a soap [opera]” (Interview 9, Clinical lead)*

*“For young people, peer educators would be very useful…younger people talking to younger people about pre-pregnancy counselling and pre-pregnancy health and sort of health during the pregnancy, and you know what to avoid, what you need to do well, you know, to keep yourself healthy as possible. But coming from peer educators rather than from midwives or GPs, might be more powerful” (Interview 12, Clinical lead).*

*“…you don't have to do it in a GP Surgery, just general education so. whether it's women's magazines. websites, there's loads of things like. I mean mums.net is for those who are pregnant but they're always then going to go on to their second pregnancy, or their third pregnancy, so talking about things there. Umm. there must be a whole forum, I don't know all the social networking sites for young people, but. I'm sure face book or something or whether it's bebo or something like that, in schools. There're a whole host of places where you can put information out there” (Interview 18, GP).*

*“Think you have to really empower patients and a lot of work has to be done with patients, empower them to actually seek the information, in terms of opportunistic giving out of information within the health setting, it's how do you energise and enthuse the frontline staff to do it” (Interview 7, Clinical lead).*

Barriers to providing preconception care included women having unplanned pregnancies, lack of knowledge and interest among health professionals and constrained resources, especially in general practice. Raising awareness in people of reproductive age, improving knowledge and confidence through training of health professionals and providing financial incentives for delivery of preconception care in general practice were seen as necessary to improve current provision.

## Discussion

### Main findings

The USA Centers for Disease Control defines preconception care as ‘a set of interventions that aim to identify and modify biomedical, behavioural and social risks to a women's health or pregnancy outcome through prevention and management’ [21]. It is worth noting that this definition does not include men, although their preconception influence on pregnancy outcomes is increasingly recognised [13].

In this study, awareness of preconception health issues was generally low among women and health professionals. The high level of pregnancy planning contrasts with low levels of information acquired about pre-pregnancy health and low uptake of folate, even in women with a poor obstetric history or relevant medical condition. However, we found that the three months before pregnancy was a time when women who smoked cigarettes or drank alcohol were quite likely to cut down or quit these risk behaviours. Furthermore, women who received advice from a health professional before pregnancy were more likely than other women to adopt positive behaviour change *before* pregnancy, particularly taking folic acid and eating a healthier diet. For these reasons, our study presents good evidence to counter widely held perceptions that pregnancy planning is uncommon so there is little to be gained from targeting the preconception period. Rather it points to the need for more effective preconception health promotion to women with greater engagement and training of health professionals.

### Strengths and weaknesses

The strengths of this study are the combination of qualitative and quantitative data, the high response rate and collection of data before the outcome of the pregnancy was known. The high response rate may reflect the face-to-face recruitment and interest in the topic, or perhaps long waiting times when attending the antenatal service. We also used a more robust measure of pregnancy planning than most other studies. The London Measure of Unplanned Pregnancy [1] is a simple 6-item questionnaire with established psychometric properties that scores the ‘plannedness’ of a pregnancy from 0 (most unplanned) to 12 (most planned). It is valid for a current or recent pregnancy (within 12 months). The LMUP represents a significant methodological advance over other; often binary (yes/no) measures of pregnancy planning that are too blunt to capture the reality for most women.

Weaknesses include retrospective reporting of pre-pregnancy behaviours with the potential for social desirability bias (e.g. over-reporting quitting smoking). The significant association between health professional input and preconception behaviour change could be explained by reporting bias and/or confounding, that is, if women who receive input from health professionals are more likely to report and/or adopt health pre-pregnancy behaviours irrespective of any input received. However, the ‘dose effect’ of health professional advice on changing to a healthier diet and taking folic acid that remained after adjusting for confounding factors (age, ethnicity, education, previous live birth, medications, previous miscarriage/stillbirth, and LMUP score) suggests that input from health professionals can have a positive and independent impact on pre-pregnancy behaviours. A randomised trial of pre-pregnancy advice from health professionals would provide stronger evidence for or against this interpretation.

### Findings in relation to other studies

Much of the literature on preconception health and care derives from research *during* pregnancy that shows the adverse effects of various medical conditions and lifestyle behaviours during pregnancy on birth outcomes e.g. obesity, diabetes. Recent studies (from the USA, Canada and Ireland) that asked women about their pre-pregnancy health behaviour are consistent in showing that women with unintended pregnancies are less likely to take folic acid or other micronutrient supplements before conception and more likely to participate in unhealthy behaviours, such as smoking, being exposed to second-hand smoke, drinking alcohol, and using illicit drugs in the pre-pregnancy period [24], [25], [7], [11]. In all of the studies, these associations remain after adjustment for the socio-demographic characteristics associated with unintended pregnancy itself.

Studies of preconception health are seldom carried out before conception because of the difficulty of identifying women who are planning a pregnancy and likely to become pregnant within a reasonable time frame. The UK Southampton Women's Study, which recruited 12,445 non-pregnant women aged 20–34, is the only study to have followed women to pregnancy, if it occurred [20]. Of the 238 women who became pregnant within three months of recruitment, 23% had said they were not anticipating this event (unplanned pregnancy group) leaving the remainder who were in some sense planning a pregnancy. Nearly half (48%) of the latter group were taking folic acid at recruitment, but only 3.3% were following recommendations for folic acid and alcohol intake. The authors concluded that only a small proportion of women planning a pregnancy follow recommendations for nutrition and lifestyle [19]. Three studies from the USA report similar findings [16], [35], [8], [19]. All three recruited large samples and asked non-pregnant women about their current pregnancy intentions. In their Californian study, Green-Raleigh et al., found that women planning pregnancy in with the next year were less likely to report smoking, more likely to report taking a multivitamin regularly, and more likely to have seen a health professional in the last year than women planning a pregnancy more than one year in the future [16]. However, there was little difference in alcohol consumption. Of the women planning a pregnancy, 8.2% were smokers, 55.3% drinking alcohol, and only 55.3% taking a multivitamin. In their analysis of the US-wide Behavioural Risk Factor Surveillance System, Xaverius et al found that women intending pregnancy were much the same as other non-pregnant women with regards to alcohol use, binge drinking, heavy drinking, and smoking, but much more likely to be taking folic acid (72%) [35]. Chuange et al reported very similar findings from a population-based cohort study in Central Pennsylvania [8]. Women intending to become pregnant differed little from other non-pregnant women in terms of their alcohol use, smoking, fruit and vegetable consumption, and physical activity, but were significantly more likely to be taking folic acid. As in the Southampton Women's Study, the authors concluded that there was little evidence that women intending to be pregnant were more likely to engage in positive health behaviours.

The high level of pregnancy planning in our study is in keeping with other data from the UK when LMUP scores are compared across similar age groups. Use of the LMUP in a number of studies shows that around two thirds of pregnancies leading to births in the UK are planned [22], [33]; therefore a majority of women who give birth are in a position to benefit from the direct targeting of pre-pregnancy care.

Other studies have reported low awareness among women or reproductive age about folic acid and the conditions (neural tube defects) that it can prevent [3]Our study indicates that engagement between women and health professionals about preconception health and care is often lacking leading to multiple missed opportunities to improve maternal and child health. There is specific guidance for professionals on pre-pregnancy care relating to conditions such as diabetes, epilepsy and obesity [29], [30], [27], [17],but more general guidance on pre-pregnancy health and care [28]does not seem to be reaching the right settings (e.g. in primary care). Guidelines alone are not sufficient to change professional behaviour. Preconception health does not fall neatly within a particular medical specialty and despite guidance there is no standardised provision of care in the UK which may explain why responsibility for providing preconception care appears confused and delivery patchy. Another reason may be the lack of studies showing clear benefits of interventions starting *before* pregnancy on birth outcomes, or how to implement such interventions effectively. Such studies are challenging as they need to identify women before conception.

A recent study of barriers to the implementation of preconception care by general practitioners in Australia [23]concluded that lack of time was the biggest barrier. Other barriers were similar to those reported here including women not presenting at the preconception stage, competing preventive care issues, the cost associated with extending consultations to include preconception care and the lack of appropriate resources. Perceived facilitators to delivery of care included checklists, patient leaflets, waiting room posters and the availability of preconception care consultations.

### Wider implications and future research

Awareness of preconception health issues, pregnancy planning and uptake of interventions before pregnancy care are related but distinct issues. All three are required to improve preconception health and pregnancy outcomes. In our study, women who received health professional input did not have greater educational attainment than women with no health professional input; they were more likely to have a relevant medical condition, and more likely to adopt positive behaviour changes. Together these findings suggest that focusing on pre-pregnancy intervention by health professionals would not merely benefit the ‘worried well’ or women with specific medical disorders, but could be an effective approach to addressing important health inequalities relating to smoking, alcohol and other risk behaviours. To strengthen the evidence base for preconception health, future research should focus on evaluating how to implement preconception care effectively and the impact that has on pregnancy and birth outcomes.

## Supporting Information

Appendix S1 COREQ checklist.
(DOCX)
