# Supplementary material for: How Do Women Prepare for Pregnancy? Preconception Experiences of Women Attending Antenatal Services and Views of Health Professionals
Source: PLoS One. 2014 Jul 24;9(7):e103085. doi: 10.1371/journal.pone.0103085 (PMC4109981; doi:10.1371/journal.pone.0103085)
Supplement: Appendix S1 — COREQ checklist. (DOCX) [file pone.0103085.s001.docx]

Appendix S1: COREQ checklist

| **Domain 1: Research team and reflexivity** | |
| --- | --- |
| 1. Interviewer/facilitator | Four interviewers carried out the interviews:, EH – 7, OO – 7 interviews, and VM (see acknowledgements) – 4, JSh - 3. |
| 1. Credentials | JSh and EH were professional researchers (PhD and MSc respectively). JSh also has a midwifery and nursing background. OO and VM were medical doctors at registrar level. |
| 1. Occupation | All interviewers were employed by, or under contract to, UCL. |
| 1. Gender | All interviewers were female. |
| 1. Experience and training | JSh and EH were experienced qualitative interviewers, with OO and VM new to qualitative research. GB and JSh led qualitative training for the research team. JSh supervised OO and VM in their early interviews. |
| 1. Relationship established | No prior relationship between the interviewers and interviewees existed. |
| 1. Participant knowledge of the interviewer | Potential interviewees were informed about the study by email, with the study information sheet approved by the research ethics committee attached. Potential participants knew that the study was funded by the Department of Health and carried out by UCL researchers. |
| 1. Interviewer characteristics | All interviewers, and members of the wider research team, had pre-existing interests in women’s reproductive health and pregnancy. JSh had a long-standing interest in pre-pregnancy care. |
| **Domain2: Study design** | |
| 1. Methodological orientation and theory | This qualitative portion of this study was informed by the principles of “qualitative description” (Sandelowski 2000, Neergaard et al 2009) where qualitative methods are used to gain firsthand knowledge of participants’ experience and perceptions of a topic. |
| 1. Sampling | Purposive sampling: health professionals from sexual and reproductive health, midwifery, obstetrics and gynaecology, and general practice were identified and contacted. The research team ensured that the final interview sample included at least one clinical lead from each of the nine governmental regions. |
| 1. Method of approach | Initial contact was by email with information about the study. |
| 1. Sample size | Twenty interviews were aimed for, 21 were achieved: the choice of sample size was pragmatic, based on prior experience of similar qualitative research. |
| 1. Non-participation | Fifty potential participants were contacted, 19 did not respond. Of the 31 responders, 5 declined, mainly because of lack of time. Of the 26 potential participants who were willing to be interviewed, 21 interviews were carried out in the available time. |
| 1. Setting of data collection | Interviewees were in their work or home location at the time of the telephone interview. |
| 1. Presence of non-participants | To our knowledge, non-participants were not present with the interviewees during the telephone interviews. |
| 1. Description of sample | Information about professional background and gender were collected. |
| 1. Interview guide | Topics (described in main text) with prompts. |
| 1. Repeat interviews | There were no repeat interviews. |
| 1. Audio/visual recording | Interviews were audio-recorded. |
| 1. Field notes | Brief field notes were kept by interviewers. |
| 1. Duration | Interviews lasted between 15 and 40 minutes, with an average of 30 minutes. |
| 1. Data saturation | Under the principles of “qualitative description” there is no assessment of data saturation as there is no process of theory development. |
| 1. Transcripts returned | Transcripts were not returned to interviewees. |
| **Domain 3: Analysis and findings** | |
| 1. Number of data coders | JSh was the primary coder, with further coding by OO. Coding consistency was co-checked by the two coders. |
| 1. Description of coding tree | Codes represented distinct viewpoints on each topic. |
| 1. Derivation of themes | Themes were closed related to the subject matter of the topic guide. |
| 1. Software | NVivo 10 |
| 1. Participant checking | There was no formal process of participant checking of research findings. |
| 1. Quotations presented | Yes, with professional background identified. |
| 1. Data and findings consistent | We have attempted to present our findings in a clear manner, consistent with the data collected |
| 1. Clarity of major themes |  |
| 1. Clarity of minor themes |  |
